# Bioleaching and chemical leaching of magnesium from serpentinites (Zlatibor Mt. ophiolite massif, Serbia) with potential application in mineral carbonation process for CO_2_ sequestration

**DOI:** 10.3389/fmicb.2025.1646341

**Published:** 2025-09-22

**Authors:** Srđan Stanković, Nebojša Atanacković, Jana Štrbački, Sabina Kovač, Kristina Šarić, Đurica Nikšić, Axel Schippers

**Affiliations:** ^1^Faculty of Biology, University of Belgrade, Belgrade, Serbia; ^2^Faculty of Mining and Geology, University of Belgrade, Belgrade, Serbia; ^3^Federal Institute for Geosciences and Natural Resources, Hannover, Germany

**Keywords:** serpentinites, carbon sequestration, magnesium extraction, leaching, bioleaching

## Abstract

Magnesium (Mg) extraction via leaching of serpentinite rocks from of the ultramafic massif of Zlatibor Mountain, Serbia, could be applied in mineral carbonation processes for permanent CO_2_ sequestration. Four different Mg leaching approaches were experimentally tested: (1) sulfuric acid leaching in chemical reactors, (2) bioleaching with the sulfur-oxidizing bacteria *Acidithiobacillus thiooxidans* in bioreactors, and (3) simulated heap leaching of coarse rock particles in percolators with both, chemical and biological methods. Experimental results show that bioleaching in bioreactors and leaching with sulfuric acid were most efficient with Mg leaching degrees of 95 ± 3.9% and 92 ± 2.8%, respectively. Bioleaching and leaching with sulfuric acid in percolators was less efficient, with Mg extractions of 16 ± 1.4% and 73 ± 2.9%, respectively. This study proposes new pathways for the development of cost-effective, scalable solutions for mitigating climate change using abundant ultramafic rock resources.

## Highlights

- Serpentinites from Serbia were used for Mg extraction via bioleaching and chemical leaching in stirred tank reactors and column percolators.
- Chemical leaching and bioleaching in tank reactors achieved > 90% Mg recovery.
- Leaching with H₂SO₄ and bioleaching in percolators extracted 73% and 16% Mg, respectively.
- Heap leaching with H₂SO₄ using coarse rock particles might be the optimal route for application.

## 1 Introduction

Mineral carbonation of magnesium-rich rocks is a naturally occurring process that captures CO_2_ from the atmosphere and produces magnesium carbonates, a stable and metastable group of minerals capable of storing carbon dioxide for long periods of time (hundreds and thousands of years). A wide variety of magnesium-rich rocks occur in nature. These include basalts, gabbros, a variety of peridotites, metamorphic rock series such as serpentinite and greenschists (chlorite-bearing rocks), and weathered Mg-rich laterites. Ultramafic rocks, peridotites (e.g., lherzolites, harzburgites, and dunites), are the Mg-rich rocks with the highest magnesium content (45–35 wt% MgO), followed by kimberlites (diamond-bearing volcanic rocks) with about 30 wt% MgO and gabbros with about 10 wt% MgO. The low-grade metamorphism of these rocks produces serpentinites and greenschists with Mg contents of 35–5 wt% MgO, depending on the type of metamorphosed rock (Haldar, 2020).

The region of the Balkan Peninsula, as part of the global Alpine-Himalayan collision belt, is known for its large peridotitic massifs that crop out along two large NW-SE trending ophiolitic belts of Jurassic age (e.g., Robertson et al., 2009; Schmid et al., 2020). During the evolution of the Tethyan Ocean, ultramafic and mafic rocks were metamorphosed and significant amounts of serpentinites were formed by almost complete serpentinization of ultramafic rocks. Serpentinites can be formed wherever ultramafic rocks are infiltrated by carbon dioxide-poor hydrothermal fluids. The final mineral composition of serpentinite is usually dominated by lizardite, chrysotile and antigorite (minerals of the serpentine subgroup), remnants of olivine and pyroxenes, spinels, and variable amount of magnetite. Lizardite, chrysotile and antigorite all have approximately the chemical formula ${\mathrm{Mg}}_{3}{\mathrm{Si}}_{2}O_{5}{\left(\mathrm{OH}\right)}_{4}$ but differ in minor components and crystal structure.

Exposure of peridotites and serpentinites to air and water results in their mineral carbonation, i.e., the precipitation of magnesium and calcium carbonate minerals. It is estimated that global natural weathering of ultramafic rocks contributes to the sequestration of 2.4 Gt of carbon dioxide per year (Bullock et al., 2021). The most famous example is a large peridotite massif in Oman, which covers an area of almost $1\times 10^{5}$ km^2^ and sequesters approximately $1\times 10^{5}$ tons of CO_2_ per year through a natural passive carbonation process (Kelemen et al., 2020). Serpentinites and dunites are the rocks with the highest estimated theoretical carbon sequestration potential, 722 kg and 690 kg CO_2_ per ton of rock, respectively. Among minerals, forsterite (786 kg CO_2_/t) and lizardite (663 kg CO_2_/t) have the highest carbon sequestration capacity (Bullock et al., 2021).

Enhanced weathering is a carbon dioxide removal (CDR) technology based on accelerated rock dissolution that sequesters CO_2_ through alkalinity production (Bullock et al., 2022). The essence of this strategy is to accelerate the process of rock dissolution, release Mg ions into solution, and then create the alkaline conditions necessary for the crystallization and precipitation of magnesium carbonates. This process can be illustrated by the chemical weathering of serpentinite minerals. These minerals dissolve in acidic environments according to the following chemical equation (Bullock et al., 2022):


(1) $${\mathrm{Mg}}_{3}{\mathrm{Si}}_{2}O_{5}{\left(\mathrm{OH}\right)}_{4}+6H^{+}\to 3{\mathrm{Mg}}^{2+}+2{\mathrm{SiO}}_{2}+5H_{2}O$$

In alkaline conditions, magnesium ions react with carbonate ions forming hydrated magnesium carbonates such as hydromagnesite that precipitates from the solution:


(2) $$5{\mathrm{Mg}}^{2+}+4{\mathrm{CO}}_{3}^{2-}+2{\mathrm{OH}}^{-}+5H_{2}O\to {\mathrm{Mg}}_{5}{\left({\mathrm{CO}}_{3}\right)}_{4}{\left(\mathrm{OH}\right)}_{2}\times 5H_{2}O$$

Geological carbon storage in, e.g., magnesium-rich rocks according to Kelemen and Matter (2008) is limited by a:

a) Slow dissolution rate of Mg-bearing minerals by a slightly acidic meteoric water which limits chemical Equation 1.
b) Low concentrations of CO_2_ in the atmosphere which limits chemical Equation 2.

Bodenan et al. (2014) created a map of the global distribution of ultramafic massifs and mine tailings suitable for mineral carbonation, located less than 300 km from large industrial CO_2_ emitters. Unlike countries such as Canada and Australia, where magnesium-rich rocks and mine tailings are often located hundreds or even thousands of kilometres away from any industrial source of CO_2_, in Serbia—and the Balkans in general—magnesium-rich ultramafic rocks suitable for mineral carbonation are often located in close proximity to major industrial CO₂ sources. The most economical way to transport CO₂-rich flue gas from industry is by pipeline. For distances up to 300 km, recompression is not required, whereas longer pipelines need recompression stations (Bodenan et al., 2014). Many magnesium-rich rock deposits and mining tailings in this region are situated well within this 300 km threshold, creating a strong opportunity to develop facilities for CO₂ capture and storage from industrial flue gases.

The Zlatibor Mountain region, for example, lies less than 300 km from major Serbian CO₂ emitters such as coal-fired power plants and cement factories. That large ultramafic massif represents a promising location for development of carbon capture and storage infrastructure.

A large-scale mineral carbonation process in this context would involve extracting magnesium from ultramafic rocks and converting it into stable magnesium carbonate minerals, which can sequester CO₂ over geological timescales. Given the close proximity of major CO₂ sources, the availability of CO₂ is unlikely to be a limiting factor; the main challenge is optimizing magnesium extraction from local rocks.

We experimentally investigated two approaches for magnesium extraction:

1. Comparison of leaching Mg with sulfuric acid in beakers (chemical reactors) and bioleaching in stirred bioreactors by biogenic sulfuric acid using finely crushed serpentinite rocks.
2. Heap leaching simulation in percolators using sulfuric acid and sulfur-oxidizing bacteria in two parallel experiments, using coarsely crushed serpentinite rocks.

Experiment 1 aimed to compare direct chemical leaching with 1 M sulfuric acid against bioleaching using approximately 1 mol of elemental sulfur (32 g). Theoretically, complete bacterial oxidation of 1 mol of sulfur produces 1 mol of sulfuric acid. While bioleaching proceeds more slowly, it could offer significant cost savings—critical for making large-scale CO₂ sequestration economically viable.

Experiment 2: simulated heap leaching, which requires coarse rock particles, thus avoiding the high energy costs of fine grinding necessary for chemical reactors or bioreactors. Although larger particle sizes generally reduce leaching efficiency, heap leaching offers lower capital and operational costs. The optimal technology choice must therefore balance extraction efficiency with energy and cost considerations.

To the best of our knowledge, this is the first study to investigate serpentinites bioleaching in bioreactors, providing a novel approach to coupling magnesium extraction with sustainable CO₂ removal strategies.

## 2 Materials and methods

### 2.1 Rock sampling and mineralogical and chemical analyses

Approximately 50 kg of serpentinites were collected from the ultramafic massif of Mountain Zlatibor in Western Serbia. Ultramafic rocks are readily available since the Mt. Zlatibor massif is constituted mostly of serpentinites. The serpentinites were collected from the road cut at the Dobroselica locality (Municipality of Čajetina). Many serpentinite rocks are excavated in this area for construction of roads, houses and hotels, since Zlatibor is a popular touristic area, making them available for application in carbon sequestration process. The samples were hand-excavated to avoid rocks that are directly exposed at the surface. The rock samples were crushed to a grain size of maximal 2 mm for serpentinite dissolution experiments in reactors and to a particle size between 1 and 3 cm in diameter for percolator experiments. The mineral phase composition of the samples was determined by powder X-ray diffraction (PXRD). The data were collected on a Rigaku SmartLab X-ray diffractometer using Bragg–Brentano geometry (CuKα radiation, 40 kV, 30 mA) at room temperature. The scan range was from 2 to 65° 2θ with a scanning speed of 10°/min while step size was 0.01°. Rigaku PDXL 2 software (Rigaku, 2007) with PDF-2 database (Gates-Rector and Blanton, 2019) was used for mineral phase identification. Solids were dissolved via microwave treatment in acid mixture (HF, HNO_3_, HCl) and consecutive element concentration measurement with ICP-OES (Thermo Scientific iCAP PRO).

Optical microscopy investigations were performed by polarization transmitted-light microscopy using a Leica microscope—type DMLSP coupled with a digital camera FC290HD and software LAS V4.1. The rock sample was cut to around 0.02 mm thickness and embedded into Canada-balsam. SEM-EDS analyses carried out at UB-FMG using a JEOL JSM-6610LV SEM coupled with an EDS detector Xplore 30 by Oxford Instruments and software AZtec 6.0 SP2 by Oxford Instruments. Samples were cut and polished, and the polished sections were covered by carbon to provide their electroconductivity, using a sputter coating instrument Leica BALTEC-SCD-005. The measurements were done under high vacuum and an acceleration voltage of 20 kV. Both internal and external standards were used for chemical analyses. As the analyses were done on polished sections, they can be regarded quantitative. The detection limit for most elements is around 0.1 wt%.

### 2.2 Experimental procedure

Sulfuric acid experiments in a beaker (chemical reactor): 500 mL of 1 M sulfuric acid and 100 g of rock crushed to 2 mm were used. The experiments were carried out in a 1 L beaker placed on a magnetic stirrer (LLG Unistirrer 7 pro) by applying a stirring speed of 600 rpm at a constant temperature of 30 °C controlled by a temperature probe immersed in the solution. The duration of the experiment was 24 h. Liquid samples for ICP-OES analysis were collected at 15, 30, 60, 120, 240 and 1,440 min and filtered through 0.22 μm PTFE syringe filters (Sartorius, Germany) before dilution 10x in distilled water. pH was measured using a pH electrode with internal silver/silver chloride reference electrode. Experiments were performed in triplicate.

Bioreactor experiments: Experiments were conducted in three parallel 2 L bioreactors (Fermac 310/60, Electrolab, Tawkesbury, United Kingdom) with a working volume of 1.5 L and a constant temperature of 30 °C maintained by a temperature controller, using a temperature probe to measure temperature and heating belt for heating of the glass reactor. The bioreactors were inoculated with a pure culture of the mesoacidophilic sulfur oxidizer *Acidithiobacillus thiooxidans* DSM 9463. Prior to inoculation, bacteria were grown in 300 mL shake flasks containing 150 mL of bacterial culture in basal saline at pH 3 (Wakeman et al., 2008) supplemented with 1% (w/v) elemental sulfur. Flasks were placed on an orbital shaker at 120 rpm for 7 days at a constant temperature of 30 °C. Bioreactors containing 1,350 mL of basal salt solution at pH 2.5 and 32 g of sulfur were inoculated with 150 mL of active bacterial culture containing approximately 10^8^ cells/mL, determined by counting of bacterial cells using Thoma counting chamber and phase contrast microscope. In order to produce sufficient bacterial biomass, the bioreactors were run for 3 days prior to the addition of the rock sample. After 3 days of incubation, when the pH of the solution was lowered to 0.8 and cell density in each bioreactor reached approximately 10^8^ cells/mL, the first batch of rock material was added to bioreactors as it was previously done for laterite bioleaching experiments (Stanković et al., 2022). Each bioreactor was fed with 15 g of crushed rock daily for 7 days until the total mass of solid material added to the bioreactors reached 105 g. This approach was chosen because, due to the substantial consumption of acid, an immediate addition of 105 g of material would prevent the establishment and maintenance of a low pH of the solution required for survival of acidophilic bacteria and efficient dissolution of serpentinites. It was not necessary to add base by automatized pH control unit (Fermac 360, Electrolab, United Kingdom) in order to maintain the pH between 0.8 and 1.5 throughout the 14-day experiments, since dynamic equilibrium between acid production by bacteria and acid consumption by rock material was established.

Percolator experiments: four glass columns (50 cm high and 5 cm in diameter) were filled with 100 g of crushed rock. Two percolators were fed with 500 mL of 1 M sulfuric acid using Reglo ICC peristaltic pumps at a flow rate of 2 mL/min in recirculation mode. For the bioleaching experiment, a bacterial culture of the sulfur-oxidizing bacteria *At. thiooxidans* was prepared in a bioreactor as described in the previous section; 1,000 mL of bacterial culture were equally distributed to two 1 L borosilicate glass bottles, each containing 500 mL of liquid and connected with two percolators. Rock particles were coated with hydrophilic “wet sulfur,” being colonized by bacteria, and placed in percolators similar to the procedure described by Stanković et al. (2024). The bacterial leaching solution was delivered by Reglo ICC peristaltic pumps at a flow rate of 2 mL/min in recirculation mode. The duration of the experiment was 21 days.

For all experiments liquid samples for pH and metal concentration measurements were collected three times per week, solid samples were analysed at the end of the experiments as described for the rock sample.

## 3 Results

The following amounts of selected chemical elements were measured for the serpentinite sample (g/kg): Si 325; Mg 130.26; Fe 51.15; Ca 9.46; Al 5.08; Ni 1.99; Na 0.1. Mineralogical analysis of the sample by XRD (Table 1; Supplementary Figure 1) revealed typical serpentine mineral phases such as lizardite and chrysotile, as well as clinochlore (chlorite) and enstatite (pyroxene). More detailed mineralogical and petrographical analysis showed that the studied rock sample is lepidoblastic to blastogranular in texture and massive, locally schistose in fabric. The majority of the rock (around 70 vol%) is composed of serpentine that creates the rock matrix that encloses remnants of primary minerals – olivine, orthopyroxene, clinopyroxene and Cr-spinel (Supplementary Figures 2a,b). Olivine appears as oval relicts or cluster of relicts with high birefringence, whereas orthopyroxene is usually elongated and displays typical pyroxene cleavages. Some orthopyroxene grains exhibit exolutions of clinopyroxene along the cleavage planes, which show textural evidence of ductile deformations characteristic for tectonite peridotites (Supplementary Figure 2c). Clinopyroxene is present as rare and small (0.5–1 mm) remnants. Cr-spinel is disseminated as individual and irregularly shaped grains with characteristic dark-brownish pleochroism (Supplementary Figure 2d) in transmitted-light optical microscope. SEM-EDS analyses results of the above-mentioned minerals are given in Supplementary Table 1.

**Table 1:** Crystal phases identified in the serpentinite sample and in the solid residues after selected experiments.

Mineral phase	Serpentinite sample	Sulfuric acid beakers leaching residue	Bioreactors leaching residue
Lizardite	x	x	x
Chrysotile	x	–	–
Clinochlore (chlorite)	x	x	x
Enstatite (pyroxene)	x	x	x
Augite (pyroxene)	–	x	x
Magnesiohornblende (amphibole)	–	x	x
Hexahydrite (magnesium sulfate)	–	x	x
Gypsum	–	x	–
Sulfur (added to the bioreactor experiment)	–	–	x

Crystallographic analysis of the solid residues after sulfuric acid dissolution in beakers and bacterially mediated mineral dissolution in bioreactors shows that chrysotile (present in the serpentinite sample) disappeared and four new crystal phases were detected (Table 1; Supplementary Figures 3, 4).

**Figure 1:**
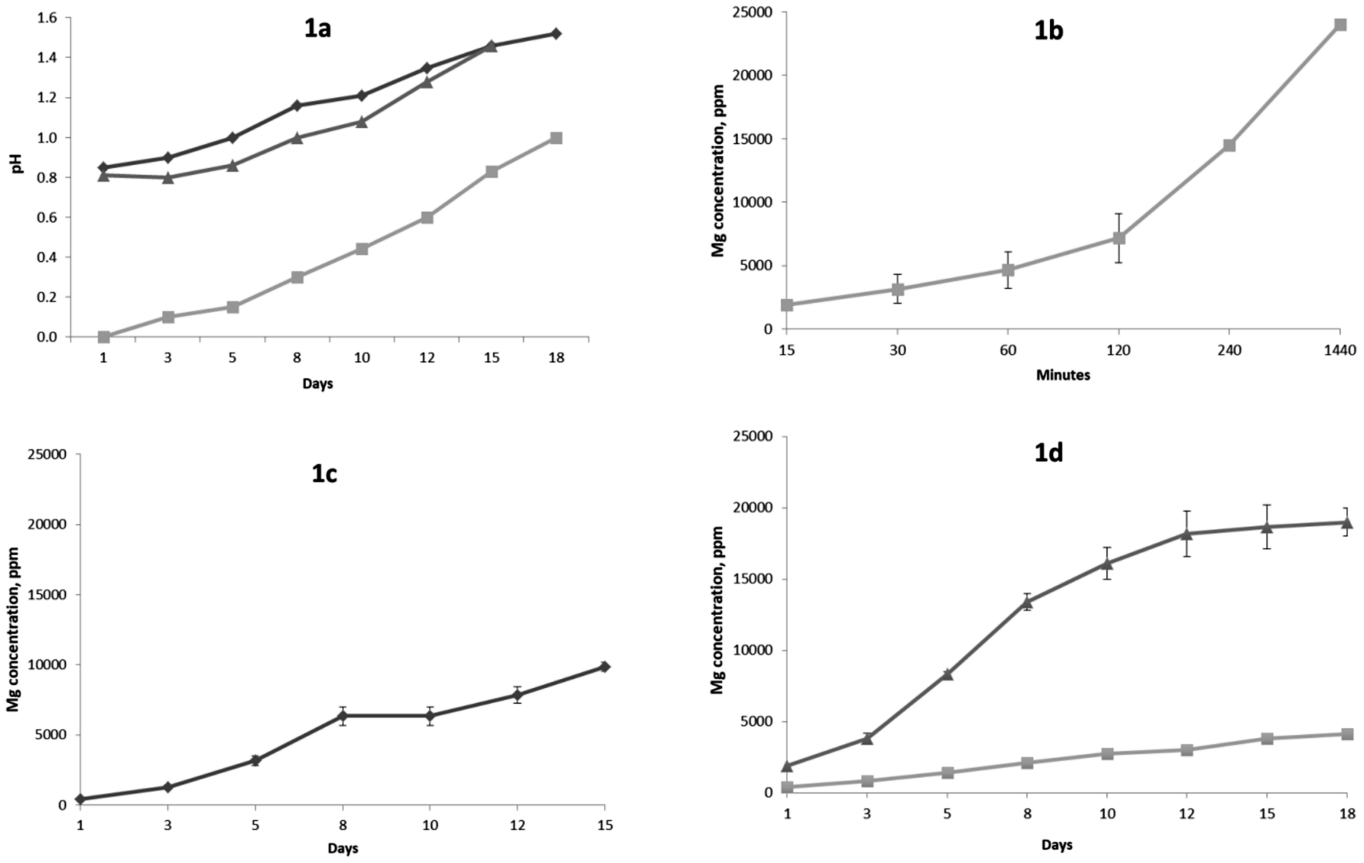
Changes in pH during experiments in bioreactors and percolators. **(a)** Rhombus – bioleaching in percolators, square – leaching with sulfuric acid in percolators, triangle – bioleaching in bioreactors; **(b)** Changes in Mg concentration during leaching with 1 M sulfuric acid in beaker; **(c)** Changes in Mg concentration during bioleaching experiment in bioreactors; **(d)** Changes in Mg concentrations during leaching in percolators with sulfuric acid and bioleaching with sulfur-oxidizing bacteria, square – bioleaching, triangle – leaching with sulfuric acid. Error bars represent standard deviations. If error bars are not visible standard deviations fit within size of the data points.

During the dissolution experiment with 1 M sulfuric acid in a beaker, the solution pH increased from negative values at the beginning of experiment to approximately 0.6 at the end of experiment (data not shown). Figure 1a shows changes in solution pH values during the experiments in percolators and bioreactors. Changes in magnesium concentrations during the experiments are presented in Figures 1b–d. The final concentration of magnesium in the beaker experiment with 1 M sulfuric acid reached approximately 24 g/L (24,000 ppm) corresponding to Mg extraction of 92 ± 2.8%. Final extraction values of Fe and Ca were 82.65 ± 6.9% and 28.53 ± 3.1%, respectively. Concentration of Si in final solution was approximately 110 mg/kg, which corresponds to 0.17 ± 0.06% of silica extraction.

The magnesium concentration in bioreactors reached approximately 10 g/L (10,000 ppm) corresponding to a Mg extraction of 95 ± 3.9%. Final extractions of Fe, Ca and Si were 60.29 ± 2.9%, 26.42 ± 1.4% and 0.5 ± 0.07%. A two-sample *t*-test showed no statistically significant difference between Mg extractions during bioleaching experiment in bioreactors and leaching with 1 M sulfuric acid in chemical reactors (t = 0.61, *p* = 0.59). Application of the two-sample *t*-tests comparing Mg leaching in (1) chemical reactors versus percolators and (2) bioreactors versus percolators showed that the differences in Mg extractions were statistically significant (*p* < 0.05). Final concentrations of magnesium after dissolution with sulfuric acid and microbially mediated dissolution in percolators were approximately 19 g/L (19,000 ppm) and 4 g/L (4,000 ppm), respectively, corresponding to magnesium extractions of 73 ± 2.9% and 16 ± 1.4%. Extractions of Fe, Ca and Si after sulfuric acid dissolution in percolators were 52 ± 4.7%, 10.57 ± 1.9% and 0.23 ± 0.4%, respectively. Dissolution with microbially generated sulfuric acid led to lower extractions of Fe, Ca and Si: 8.8 ± 1.1%, 5.28 ± 0.9 and 0.09% ± 0.01, respectively.

The data on extracted Mg, Fe, Ca and Si are summarized in Table 2.

**Table 2:** Mg, Fe, Ca, and Si extraction in the experiments based on concentrations in solution.

Experiment	Mg, %	Fe, %	Ca, %	Si, %
1 M sulfuric acid beakers	92 ± 2.8	82.60 ± 6.9	28.53 ± 3.1	0.17 ± 0.06
Bioreactors	95 ± 3.9	60.29 ± 2.9	26.42 ± 1.4	0.5 ± 0.07
1 M sulfuric acid percolators	73 ± 2.9	52 ± 4.7	10.57 ± 1.9	0.23 ± 0.4
Bioleaching percolators	16 ± 1.4	8.8 ± 1.1	5.28 ± 0.9	0.09 ± 0.01

## 4 Discussion

The serpentinite sample was dominated by typical serpentine group minerals such as lizardite and chrysotile, and the latter was completely dissolved during the acid leaching experiments in which Mg was efficiently extracted from the rock in most cases. The very low extraction of silica in all experiments means that the silicate framework remained intact. This finding confirms the incongruent mineral dissolution of serpentine group minerals by acid treatment: Mg^2+^ ions are released into solution due to the preferential attack of H^+^ ions on Mg-OH bonds in the crystal lattice of the serpentine mineral phases (Lacinska et al., 2016). The efficiency of magnesium extraction from serpentine minerals follows the trend: chrysotile > lizardite > antigorite (Arce et al., 2017). Consistent with the results of our study, previous studies reported almost complete magnesium extraction from serpentinite after acid dissolution, with sulfuric acid identified as the most efficient leaching agent (Teir et al., 2007; Arce et al., 2017).

The percentage of Mg extracted by biogenic sulfuric acid leaching in bioreactors was comparable to that obtained by 1 M sulfuric acid leaching in beakers and percolators, although the microbial process was significantly slower. From an economic feasibility perspective, the main products of the carbon removal process are tons of sequestered CO_2_. This “commodity” has a relatively low market value,^1^ so it is imperative to develop a low-cost process. Sulfuric acid has been shown to be efficient for extracting magnesium from ultramafic rocks at temperatures between 70 °C and 90 °C (Yoo et al., 2009; Lacinska et al., 2016). In order to keep the energy consumption low, the application of sulfuric acid at 30 °C with extended reaction time was tested. The dissolution of ultramafic rocks consumes a significant amount of acid (chemical Equation 1), and high acid consumption could make a commercial process unprofitable. Therefore, we tested Mg extraction in bioreactors using elemental sulfur as a feedstock for the sulfur-oxidizing acidophilic bacteria *Acidithiobacillus thiooxidans*. The use of elemental sulfur instead of sulfuric acid should reduce the operating costs for Mg extraction—sulfur is produced in large quantities as a by-product of oil rafination process; advantages of using sulfur instead of sulfuric acid also include lower transportation costs, simpler storage and lower risk of environmental hazards. Production of one ton of biogenic sulfuric acid requires 0.327 tons of sulfur. If we apply the qualified presumption that the price of 1 ton of sulfur is 100 USD, and the price of one ton of sulfuric acid is 165 USD, it can be calculated that the cost of raw material for biogenic production of 1 ton of sulfuric acid is only 32.7 USD.

The potential disadvantage of using chemical reactors and bioreactors for mineral carbonation is the significant capital cost of purchasing this sophisticated equipment, the operational cost of maintenance, and the need for highly skilled engineers to control and optimize the process. For this reason, we additionally tested Mg extraction using laboratory-scale columns (percolators) that simulate a “heap leaching” process that is routinely used on a commercial scale for the production of metals such as copper, zinc, nickel, cobalt, and uranium (Ghorbani et al., 2016; Petersen, 2023). Heap leaching is based on the construction of a heap that is irrigated with an acidic leaching solution; the recirculated pregnant leaching solution is collected in a pond and then transported to downstream processing for metal extraction. Power et al. (2010) tested a bioleaching approach for the extraction of magnesium from chrysotile asbestos mine tailings using small columns. They reported a maximum Mg extraction of 14.3% in columns amended with sulfur and inoculated with the sulfur-oxidizing bacteria *Acidithiobacillus thiooxidans*. This result is comparable to the Mg extraction achieved in the glass column bioleaching experiments in this study (16 ± 1.4%). Substantially lower Mg extraction during bioleaching in percolators in comparison to bioleaching in bioreactors is not surprising. Much bigger particle size in percolators gives lower surface area for chemical reactions. Also, bioreactors provide controlled conditions (aeration, temperature, stirring, pH) that promote growth and activity of the bacterial culture. On the other side, percolators were running at room temperature, without additional aeration or stirring, which affects the activity of the bacterial culture and sulfuric acid production in percolators.

## 5 Conclusion

This study demonstrates that serpentinites from the Zlatibor ophiolite massif can be effectively processed for magnesium extraction, offering a viable pathway toward CO₂ sequestration via mineral carbonation. Both chemical leaching with 1 M H₂SO₄ and bioleaching with *Acidithiobacillus thiooxidans* in stirred tank bioreactors achieved high Mg recoveries (>90%), while coarse-particle percolator leaching with H₂SO₄ yielded 73% Mg recovery. Bioleaching in percolators, although less efficient (16%), confirms the feasibility of low-cost, biologically driven processes. In contrast to the bioleaching of copper sulfide ores, where oxidation of sulfide minerals like pyrite generates sulfuric acid, bioleaching of serpentine minerals does not produce acid. The process can be engineered to consume nearly all the sulfuric acid in the leaching solution, preventing acid accumulation. Moreover, the solid residues remaining after magnesium extraction are chemically inert and pose no risk of acid mine drainage. These characteristics indicate that magnesium bioleaching from serpentinites is inherently less environmentally hazardous, offering a more sustainable alternative compared to copper recovery from sulfide ores.

The results highlight that coarse-particle heap leaching could deliver magnesium concentrations in pregnant leach solutions comparable to those obtained from fine-particle chemical or bioreactor systems, while significantly reducing comminution energy requirements and capital expenditure. Integrating such low-energy extraction methods with downstream carbonation could enable scalable, cost-effective CO₂ removal, especially when coupled to the valorisation of by-products such as magnesium carbonates. The future research direction will include experimental testing of the Mg carbonation in pregnant leach solution in pressure reactors at mild temperature, in order to produce hydrated magnesium carbonate minerals, and test their potential application as supplements for production of low-carbon cement.

## Data Availability

The original contributions presented in the study are included in the article/Supplementary material, further inquiries can be directed to the corresponding author.

## Footnotes

^1^ https://tradingeconomics.com/commodity/carbon
