# Supplementary material for: Bioleaching and chemical leaching of magnesium from serpentinites (Zlatibor Mt. ophiolite massif, Serbia) with potential application in mineral carbonation process for CO2 sequestration
Source: Front Microbiol. 2025 Sep 22;16:1646341. doi: 10.3389/fmicb.2025.1646341 (PMC12497821; doi:10.3389/fmicb.2025.1646341)
Supplement: Supplementary file 1 [file Table_1.DOCX]

## Supplementary material

### X-ray diffraction and scanning electron microscopy analyses of the original sample


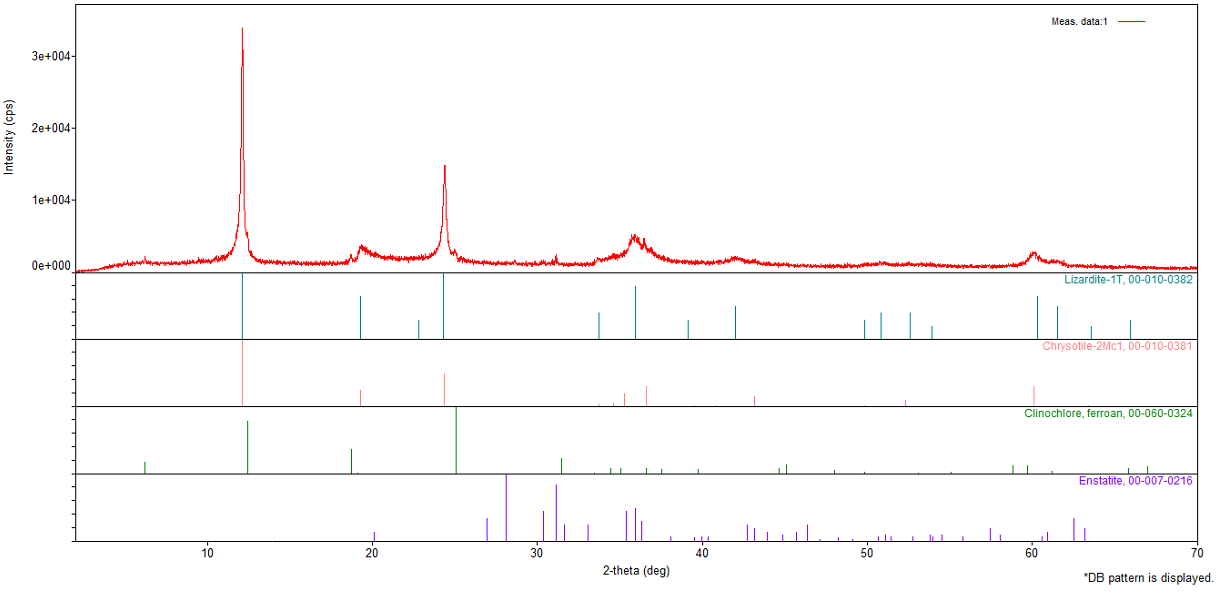


Supplementary figure 1. X-ray diffraction analysis of the original serpentinite sample.


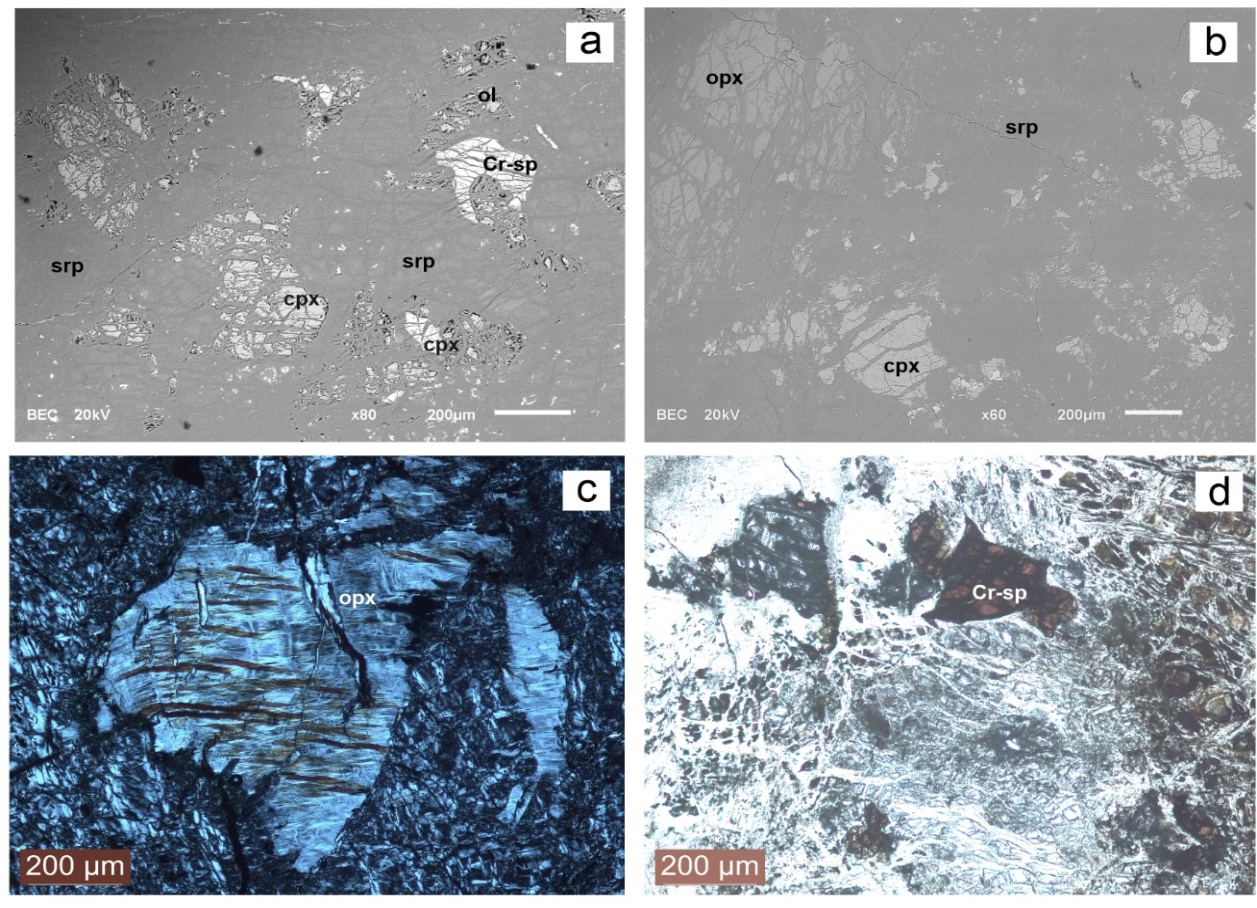


Supplementary figure 2. Petrographical characteristics of the serpentinite sample: a, b - BSE (back-scattered electron) images of the matrix composed of serpentine minerals (srp), which encloses patchy relicts of primary minerals - ol, opx, cpx and Cr-sp; c – textural evidence of ductile deformation of clinopyroxene exlolutions within an orthopyroxene porphyroblast (crossed nicols); d – individual irregularly shaped grain of dark-brownish Al-Cr-spinel (parallel nicols). Abbreviations are given in Supplementary table 1.

| Supplementary table 1. Chemical composition of the analyzed primary and secondary minerals in the serpentinite sample. | | | | | | | | | |
| --- | --- | --- | --- | --- | --- | --- | --- | --- | --- |
| Mineral / Project | ol SS2/Site 4 | opx SS2/Site 3 | Cpx-1 SS1/Site 1 | Cpx-2 SS2/Site 4 | Cr-sp-1 SS1/Site 3 | Cr-sp-2 SS1/Site 4 | Cr-sp-3 SS2/Site 2 | Srp-1 SS2/Site 2 | Srp SS2/Site 3 |
| Spectrum | No. 123 | No. 93 | No. 1 | No. 124 | No. 43 | No. 60 | No. 81 | No. 87 | No. 109 |
| SiO_2_ | 40.0 | 54.7 | 50.2 | 49.7 | 12.5 | / | 1.3 | 37.9 | 36.4 |
| Al_2_O_3_ | / | 3.8 | 3.9 | 4.3 | 25.1 | 45.7 | 21.8 | / | 0.4 |
| FeO | 10.6 | 7.1 | 2.5 | 2.8 | 19.1 | 16.1 | 25.6 | 7.5 | 11.6 |
| MgO | 49.9 | 33.4 | 16.0 | 16.2 | 20.0 | 16.0 | 8.6 | 37.1 | 34.6 |
| MnO | 0.1 | 0.2 | / | / | / | / | / | 0.1 | 0.2 |
| CaO | / | 0.6 | 23.6 | 23.2 | / | / | / | / | 0.1 |
| Na_2_O | / | / | 0.2 | / | / | / | / | / | 0.1 |
| V_2_O_3_ | / | / | / | / | 0.4 | 0.2 | 0.3 | / | / |
| Cr_2_O_3_ | / | 0.7 | 1.0 | 1.0 | 23.3 | 21.4 | 41.5 | / | / |
| NiO | 0.4 | / | / | / | / | / | / | 0.4 | / |
| ZnO |  | / | / | / | / | 0.3 | 0.7 | / | / |
| Total | 101.1 | 100.4 | 97.3 | 97.2 | 100.3 | 99.6 | 99.8 | 83.0 | 83.4 |
|  | Calc. on 4 O | Calculated on 6 O | | |  | | |  | |
| Mg# | 89.35 | 89.1 | 91.9 | 91.2 | 65.13 | 63.95 | 37.45 |  | |
| Cr# | / |  | | | 38.36 | 24.13 | 56.10 |  | |
| Fo | 89.26 |  |  |  | Abbreviations: cpx – clinopyroxene, Cr-sp – Cr-spinel, ol – olivine, opx - orthopyroxene, srp – serpentine;  Mg#=[100*MgO/(MgO+FeOt)mol] - magnesain number;  Cr#=[100*Cr2O3/(Cr2O3+Al2O3)mol] - chromium number;  Fo- forsterite component, Fa – fayalite component, En – enstatite component, Fs – ferrosilite component, Wo – wollastonite component. | | | | |
| Fa | 10.64 |  |  |  |  |  |  |  |  |
| En |  | 88.1 | 46.6 | 47.0 |  |  |  |  |  |
| Fs |  | 10.8 | 4.1 | 4.6 |  |  |  |  |  |
| Wo |  | 1.1 | 49.4 | 48.4 |  |  |  |  |  |

### X-ray diffraction analysis of the solid residue after dissolution in beaker with 1M sulfuric acid


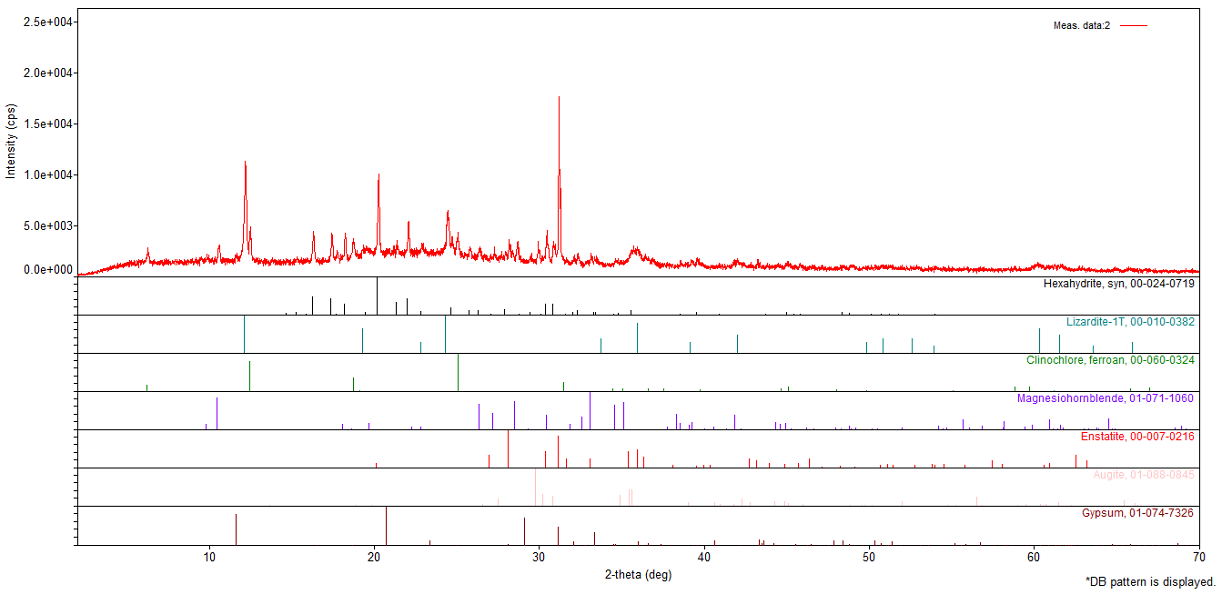


Supplementary figure 3. X-ray diffraction analysis of the leaching residue after dissolution of serpentinites in beaker with 1 M sulfuric acid.

### X-ray diffraction analysis of the solid residue after dissolution in bioreactors


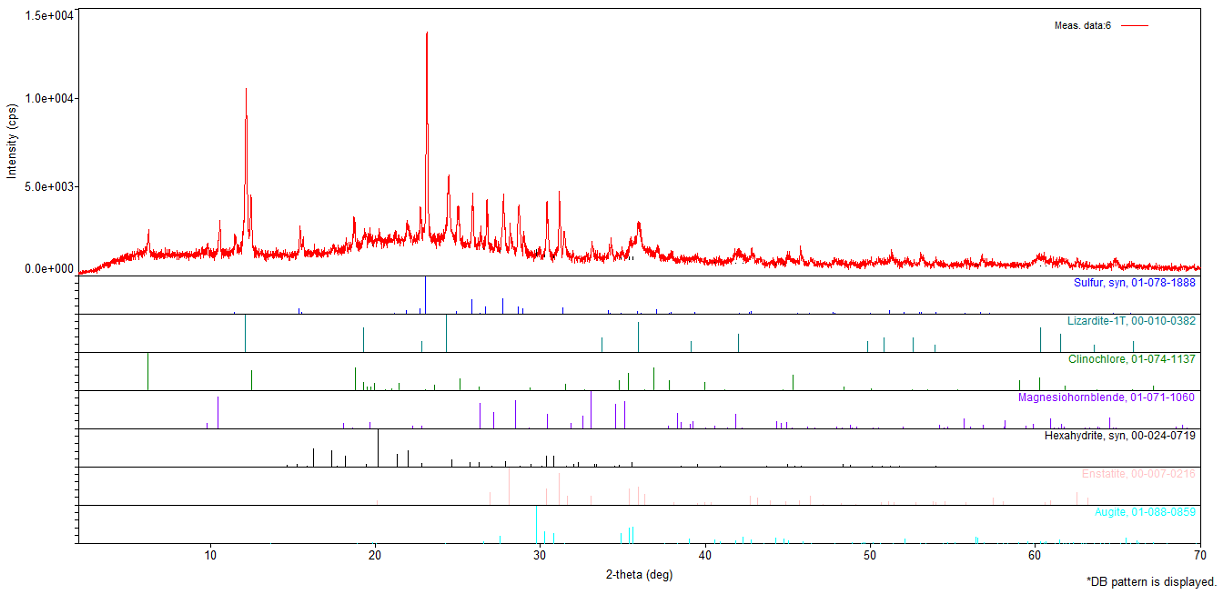


Supplementary figure 4. X-ray diffraction analysis of the leaching residue after dissolution of serpentinite in bioreactors.
